# Seeing Your Error Alters My Pointing: Observing Systematic Pointing Errors Induces Sensori-Motor After-Effects

**DOI:** 10.1371/journal.pone.0021070

**Published:** 2011-06-23

**Authors:** Roberta Ronchi, Patrice Revol, Masahiro Katayama, Yves Rossetti, Alessandro Farnè

**Affiliations:** 1 INSERM U1028, Lyon Neuroscience Research Center, ImpAct Team, CNRS UMR5292, Lyon, France; 2 University Claude Bernard Lyon I, Lyon, France; 3 Hospices Civils de Lyon, Hôpital Neurologique, Mouvement et Handicap, Lyon, France; 4 Department of Neurology, Senri Chuou Hospital, Shinsenri-higashi-machi, Toyonaka-City, Osaka, Japan; 5 Department of Community Medicine and Social Healthcare Science, Kobe University Graduate School of Medicine, Kusunoki-cho, Chuo-ku, Kobe, Japan; 6 Department of Psychology, Università degli Studi di Milano-Bicocca, Milano, Italy; 7 Neuropsychological Laboratory, Ospedale S. Luca, IRCCS Istituto Auxologico Italiano, Milano, Italy; The University of Western Ontario, Canada

## Abstract

During the procedure of prism adaptation, subjects execute pointing movements to visual targets under a lateral optical displacement: As consequence of the discrepancy between visual and proprioceptive inputs, their visuo-motor activity is characterized by pointing errors. The perception of such final errors triggers error-correction processes that eventually result into sensori-motor compensation, opposite to the prismatic displacement (i.e., after-effects). Here we tested whether the mere observation of erroneous pointing movements, similar to those executed during prism adaptation, is sufficient to produce adaptation-like after-effects. Neurotypical participants observed, from a first-person perspective, the examiner's arm making incorrect pointing movements that systematically overshot visual targets location to the right, thus simulating a rightward optical deviation. Three classical after-effect measures (proprioceptive, visual and visual-proprioceptive shift) were recorded before and after first-person's perspective observation of pointing errors. Results showed that mere visual exposure to an arm that systematically points on the right-side of a target (i.e., without error correction) produces a leftward after-effect, which mostly affects the observer's proprioceptive estimation of her body midline. In addition, being exposed to such a constant visual error induced in the observer the illusion “to feel” the seen movement. These findings indicate that it is possible to elicit sensori-motor after-effects by mere observation of movement errors.

## Introduction

The human brain has the capacity of quickly learning and adapting to environmental changes. Paradigmatic examples of such plasticity are provided by studies on sensori-motor learning of visuo-motor control under unnatural force-fields and adaptation to prism-induced displacement of the visual-field [1]–[8]. An interesting example of brain reversible plasticity is the one triggered by acting on visual space while wearing prismatic goggles [9], [10]. When a person reaches to visual targets under an optical displacement that induces a lateral (left- or right-ward) shift of the visual scene, her visuo-motor activity is initially characterised by systematic pointing errors in the direction of the optical displacement. If the final pointing error is visible, error correction takes place giving rise to sustained after-effects after prism removal: To their own surprise, subjects produce (compensatory) errors opposite to the direction of the prism deviation [7], [11]–[13]. Adaptation to altered force environments provides another example of sensori-motor plasticity. When a viscous force is experimentally applied to an otherwise voluntarily controlled arm movement, subjects learn to specifically counteract the induced force-field. Thus, the initially major action perturbation is progressively reduced by specific compensatory corrections, exerted in response to the rules acting in the novel environment [14], [15].

In the classical literature subjects develop adaptation or learning in conditions where they perceive their own error signals. Despite a wealth of studies have shown similarities between executing and observing an action (see, for review, [16]), little is known about the after-effects possibly induced by seeing someone else's error signals. Merely observing an action activates the same regions typically implied in the planning and actual execution of the same action, both in monkeys and in humans [17]–[21]. Thus, visually perceiving an action is thought to activate corresponding motor programs [22]. In agreement with this view, sensori-motor learning can occur by simple observation [23]–[25]. In a recent study, Mattar and Gribble [26] elegantly demonstrated that observing a person learning to adapt her reaching movements to a force-field environment facilitates the observer's motor learning when tested later in the same environment. Therefore, action observation is functional to learn new motor patterns and can provide information not only about what an action is for, but also on how to perform it. According to the notion that action observation can evoke an internal representation of the seen movement [27], interference effects have been found when subjects perform an action while observing an incongruent one being performed by somebody else [28]–[30]. Moreover, the same mechanisms are thought to be responsible for detecting one's own as other persons' motor errors (e.g., [31]). Finally, making and observing errors evoke similar error-related negativity on the same brain regions [32].

In the domain of prism adaptation, and consistent with the substantial similarity between executed and imagined actions [33], a pioneering study by Finke [34] showed that if subjects are required to imagine that their (real or imagined) pointing movement ends up producing a systematic error, they subsequently exhibit an after-effect that is opposite in direction to the *imagined* error. Although Finke's study demonstrated that adding an error to one's own motor imagery may produce prismatic-like after-effects, it remains presently unknown whether passive observation of somebody else's pointing errors would be sufficient to induce after-effects in the observer. Besides its own theoretical relevance as a model of plastic behaviour in the healthy human brain, another main interest for understanding the mechanisms participating (and possibly leading) to prism adaptation owes to their therapeutic implications: Prism adaptation is indeed thought of as one of the most promising techniques for the rehabilitation of left spatial neglect [10], [35]–[40].

Here we tested whether, and to what extent, the mere passive observation of pointing errors made by another person can give rise to after-effects in the observer's sensori-motor behaviour, as well as whether these are akin to those observed following prismatic adaptation. To these aims, we ran two experiments in which healthy participants did not perform any action, but observed another person making incorrect pointing movements. The rationale for the study was that compensatory correction should be elicited by simple observation of erroneous pointing actions by virtue of being, at least partially, processed via the similar processes that are implied in actual prism adaptation [36], [41]. We submitted participants to three tasks typically used in prismatic adaptation studies to assess for the presence of prisms-induced after-effects, by comparing performance before (pre) and after (post) prismatic exposure and calculating the relative shift (S) in these variables. These were, namely, proprioceptive (PS) and visual (VS) estimation of the subjective midline and visual-proprioceptive (VPS) open-loop pointing, which are all considered of as indexes of adaptation [2].

## Experiment 1

### Materials and Methods

#### Subjects

Twelve right-handed subjects (6 males, mean age: 26.6, range 22–35) with normal or corrected to normal vision participated in this study. For this, as well as for the following experiment, all participants gave their verbal informed consent to participate in the study, which was approved by the review board of the INSERM U864 ethics committee. All subjects were naive as to the purpose of the study. Only 3 out of 12 participants were aware of the usual effects induced by prisms.

#### Apparatus

**Figure 1:**
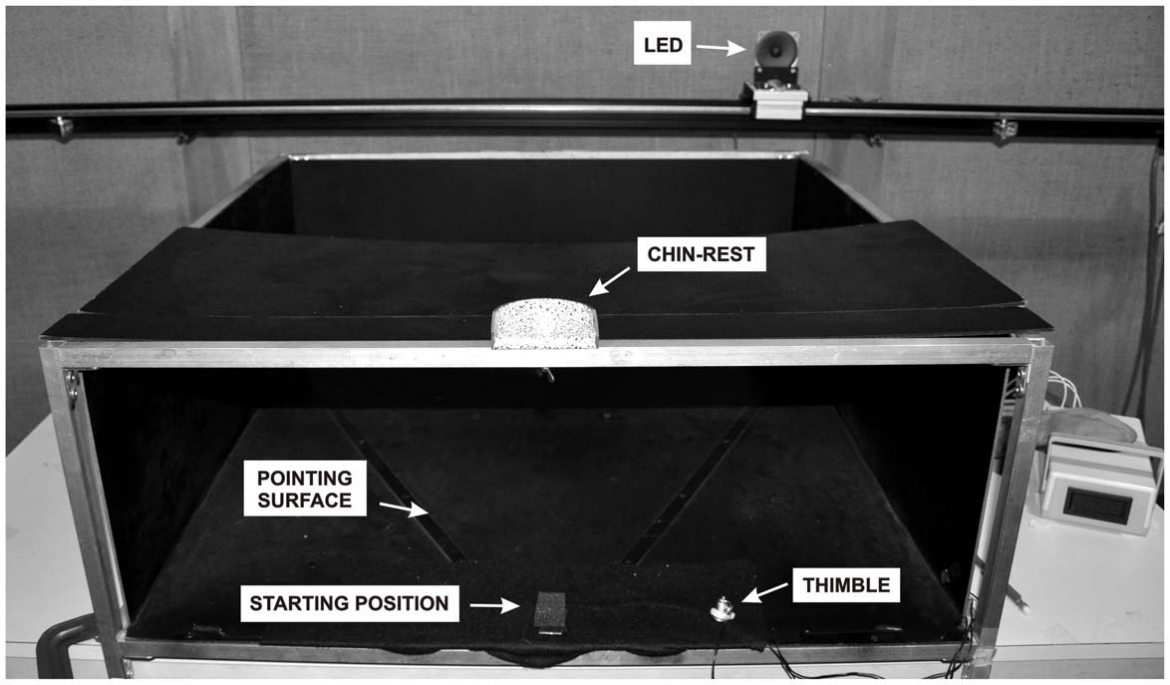
The test box.

Two custom-made experimental set-ups served different aims: a test box (identical to Rossetti et al, 1998) was used to measure and record the three dependent variables (PS, VS, VPS) before and after exposure to pointing errors; a pointing board was used to visually expose subjects to the pointing errors. The *test box* was a black wooden frame (30 cm high, 80 cm wide and 80 cm deep) opened on the side facing the subject: the lower horizontal surface was covered by electro-resistive carbon paper. The distal side facing the subject was equipped with a pulley-mounted red LED that could be moved horizontally in front of the subject (see Figure 1). The speed of the LED movement was varied between trials in order to avoid counting strategies. The resistive paper and pulley were calibrated to electronically read out the final finger position on the box lower surface, as signalled by the contacting position of a metal thimble worn on the right index fingertip. The *pointing board* was a black wooden table (50 cm×100 cm) where two filled dots (red on the left, white on the right; 1 cm diameter) were permanently visible.

#### Procedure: Pre and Post Tests

Participants sat in front of the test box in a darkened, sound-attenuated booth, with their head aligned with the body's mid-sagittal axis. The head was stabilised by having their chin on a chin-rest situated on the top of the box (see Figure 1). The subjects' right arm was placed in the box, with the right hand resting on a starting location in front of their trunk and a thimble worn on the index finger. Before and after the observation phase, the following measures of egocentric reference frames were taken:

- *Proprioceptive (P)*: with eyes closed, subjects made 10 straight-ahead pointing movements on the table surface, to indicate the subjectively estimated position of their body midline;
- *Visual (V)*: with eyes open, subjects verbally stopped for 10 times the position of a red LED, moving horizontally in front of them, when it corresponded to their body midline. The LED moved 5 times from the right to the left and 5 times from the left to the right;
- *Visual-Proprioceptive (VP)*: with eyes open, subjects made 10 pointing movements on the table surface to indicate the downward projected position of the red LED, which was placed in front of their body midline while they kept their eyes close. The arm movement was occluded from view (open loop pointing) via a wooden panel horizontally positioned on the top of the box.

For each variable, a preliminary examination of the measures revealed a stable performance across the 10 trials. Therefore, the accuracy was computed by the average of the 10 trials per task, measured in degrees of visual angle with respect to the objective body midline (corresponding to 0°): negative numbers indicated leftward-, while positive numbers rightward-located estimates. The difference between post- and pre-exposure measures was also computed to express the relative shift in estimate for each task (Visual Shift: VS, Proprioceptive Shift: PS, Visual-Proprioceptive Shift: VPS).

#### Procedure: Exposure

**Figure 2:**
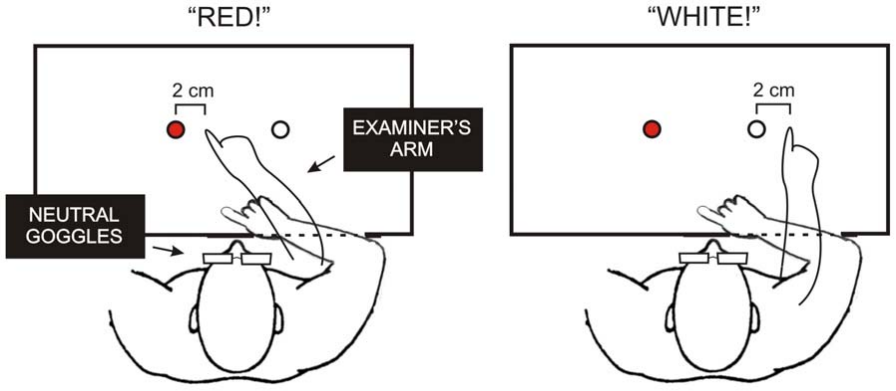
Procedure of the Experiment 1: each participant observes incorrect pointing movements performed by the examiner on the right –side of the stimulus.

Participants sat facing the pointing board where visual targets were located 20° to the left (red) and to the right (white) with respect to their mid-sagittal axis. Both the subject and the experimenter wore a white glove on their right hand: the subject's hand (unseen) was aligned with her mid-sagittal axis and placed on a support under the table with the index finger located beneath the hand of the experimenter (visible). The procedure was inspired from the Japanese traditional sketch comedy called Nininbaori, in which a person A (the ‘experimenter’) sitting behind a person B (the ‘subject’) is trying to feed B with chopsticks (http://www.english-rakugo.com/english_version/english_performance.html). The experimenter stood behind the subject with the right hand placed on the table in front of the subject's body midline, above the unseen subject's hand. The participant wore a pair of goggles fitted with neutral (non-deviating) lenses. During this phase, the subject was required to carefully observe the pointing movements performed towards the targets: the examiner called out the colour of one target (“red!” or “white!”) and right afterwards made a rapid pointing movement (entirely visible) towards it. However, the experimenter's pointing movement was “wrong” in that she voluntarily made an error on the right-side, of 2 cm on average (see Figure 2). The subject observed a total of 60 erroneous pointing movements (30 to the red and 30 to the white target, in a random order). This exposure phase lasted about 3 minutes.

#### Statistical analysis

An ANOVA was performed on the mean shift for each measure. A separate ANOVA was conducted on the mean pointing positions, in order to compare the subjects' performance in each test before and after the observation phase. Paired t-test analysis (against zero) was additionally performed for each of the three mean shifts.

### Results

**Figure 3:**
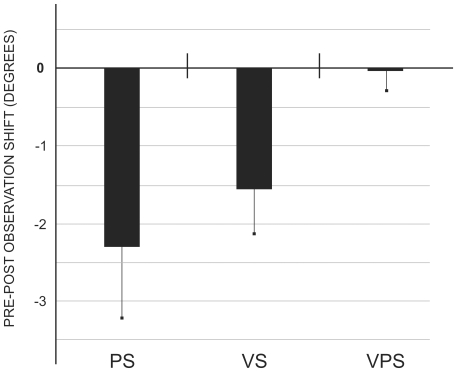
The mean (s.e.m.) proprioceptive (PS), visual (VS) and visual-proprioceptive (VPS) shift (Experiment 1). The number zero indicates the lack of differences between pre- and post-observation, negative numbers the presence of a shift to the left, positive numbers to the right.

Figure 3 shows the mean subjects' shift (difference Pre-Post observation) in each test. A repeated measure ANOVA with Shift (PS, VS and VPS) as a within-subject variable was conducted. The main effect of Shift was significant (F (2, 22) = 3.82, p = 0.038) revealing a stronger leftward deviation in PS (−2.29°) as compared to VPS (−0.01°; p<0.05), no other comparison being significant. The VS (−1.55°) tended to deviate towards the left, without differing from the other two measures. Paired t-tests confirmed that PS (t = −2.45, p = 0.032) and VS (t = −2.68, p = 0.022) significantly differed from zero (i.e., the value indicative of the absence of pre-post difference), whereas VPS did not (t = −0.03, p = 0.978).

### Discussion

These results show that observing pointing movements that deviate in their final landing position towards a relative rightward location alters the observer's estimation of the subjective body midline in the leftward (i.e., opposite) direction, both in the visual and proprioceptive modalities. No significant change was observed in the visual-proprioceptive test, also termed “total shift” [42]. It therefore seems that merely observing a rightward pointing bias, similar to the error normally executed during the initial phase of prism adaptation, does alter the subjectively sensed body midline, which is one of the most typical consequences of prism adaptation [43], [44]. Indeed, during prism adaptation, two sources of information are elaborated concerning hand position: proprioception and vision [45], [46]. The mismatch between proprioceptive and visual cues, which results in a final pointing error, is progressively compensated for by the brain and two components are thought to be implied in this process. Initially, the fast error reduction is thought to be a consequence of a strategic compensative behaviour, in which the subject uses the error feedback to voluntarily correct performance. Strategy is not sufficient, however [13], and during successive pointing trials a more automatic kind of compensation becomes established, leading to real adaptation [44]. In this respect, the results of Experiment 1 clearly show that seeing a systematic rightward error (i.e., constant around 2 cm from target) can generate a leftward after-effect. Even though the mere observation of error signals produced by somebody else does not enact exhaustively the consequences of adaptation to visual-field displacement (specifically because the motor commands and the proprioceptive feedback are absent), it seems to induce adaptation-like sensori-motor effects.

To better understand this phenomenon, and more clearly establishing whether adaptation-like after-effects follow mere passive exposure to somebody else's erroneous pointing movements, several aspects need to be further taken into account. A first point is to ascertain whether subjects appreciated the “wrongness” in the pointing movements they observed. In fact, debriefing revealed some of them did not notice anything “odd” or “wrong” because, e.g., “the experimenter pointed towards the correct target” as defined by the spoken colour. Therefore, considering the small amplitude of the naturally variable error, some subjects actually considered the seen movements as being correct. In spite of their wrong belief, they were nevertheless affected by the observation of such a ‘negligible’ error. Second, it is important to consider the potential contribution of the participant's cognitive interpretation of the seen movement, which was performed by another person, but could have induced the illusory feeling that the observer took part in the seen movement, or the moving arm could belong to the observer (i.e., agency and/or ownership illusions, see [47]–[49]). To address these issues, in Experiment 2 we introduced a questionnaire designed to ascertain the impression of 1) ‘wrongness’ about the observed movements and 2) feelings of agency and/or ownership of the seen moving arm.

As a third point, in Experiment 1 the error performed by the examiner was small and constant (except for the natural variability around the intended 2 cm of lateral deviation). This, however, is not what typically occurs during the whole prism exposure phase, when the pointing error is initially large and then decreases with the increasing number of trials. Our choice of a constant error was motivated by the fact that adaptation, as measured via visual, proprioceptive and visual-proprioceptive after-effect measures, is stronger in the initial phase of the exposure period when the error is not yet compensated for, then reaches a plateau when pointing errors are further reduced (e.g., Redding & Wallace [11]). We thus formulated the hypothesis that observing a stable error should maximise the ‘need’ to compensate. By contrast, observing an error reduction should decrease the pressure to modify the incorrect movement, so that an initial rise in compensation pressure should be followed by a wiping out of the nascent consequences. Finally, subjects' previous knowledge about the prisms' properties and the effects of prismatic adaptation could also influence the processes that are put into play during the observation phase and/or the subsequent test phase. To take into account both these additional issues, two observation phases were compared in Experiment 2. Participants observed pointing movements displaying large(r) final errors (6 cm on average) that were either constant or progressively reduced across trials. In addition, while in Experiment 1 subjects with different levels of prisms expertise were tested, in Experiment 2 participants were split in two subgroups: naives and non naives with respect to the effects of prism adaptation.

## Experiment 2

### Materials and Methods

#### Subjects

Forty-four right-handed subjects (16 males; mean age: 27.84, range: 18–55) with normal or corrected to normal vision and no history of neurological disease participated in this experiment.

#### Experimental set-up & Procedures: Pre and Post Tests

The same set-up (test and pointing boards) and procedures used in Experiment 1 were employed, unless otherwise stated.

#### Procedure: Exposure

**Figure 4:**
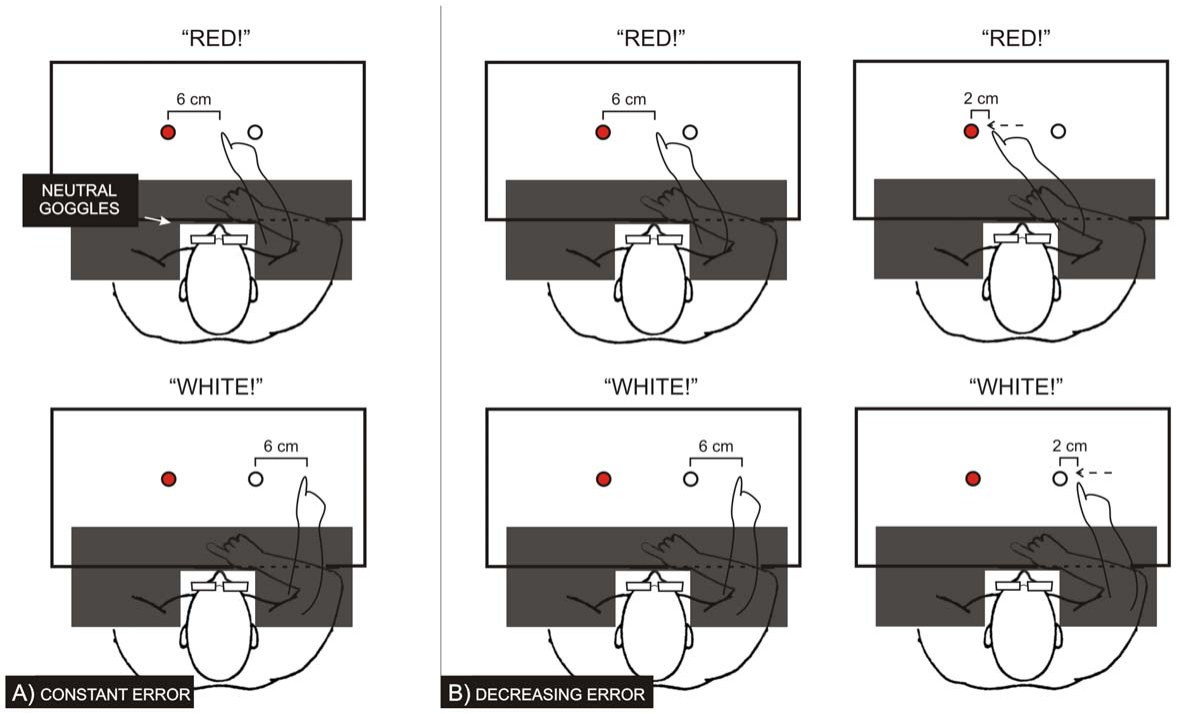
Procedure of the Experiment 2: each participant observes incorrect pointing movements performed by the examiner on the right –side of the stimulus. In the Constant Error condition (A) the error remains constant; in the Reduced Error one (B) the error is progressively reduced up to 2 cm.

At variance with Experiment 1, a black board prevented the vision of the examiner's hand when resting on the starting position (see Figure 4). In addition, a pre-recorded voice calling out targets by colour was played to start the examiner pointing movement. In a pre-experimental phase, subjects were shown what a ‘correct’ pointing movement was like: the participant did not wear the goggles and observed 20 correct pointing movements on the targets (10 to the right and 10 to the left, in a random order). Immediately after, the subject wore the goggles fitted with neutral (non-deviating) lenses and was required to carefully observe the pointing movements, which were visible for about the last two/thirds. Subjects were split into two error observation conditions (Figure 4):

- Constant Error (22 subjects: 12 naives and 10 non-naives with respect to the effects induced by prismatic adaptation): the examiner made 60 rapid movements towards the targets (i.e., 30 trails per target position), making a voluntary error on the right-side of about 6 cm. The error was constant, except for the small natural variability in the examiner's pointing movements.
- Decreasing Error (22 subjects: 12 naives and 10 non-naives, as above): the examiner made 60 rapid movements towards the targets (i.e., 30 trails per target position), making initially a voluntary error on the right-side of about 6 cm that was reduced trial by trial to leave a residual error of 2 cm from the targets.

Both exposure phases lasted about 3 minutes.

#### Procedure: Questionnaire

At the end of the experiment, a questionnaire was administered. Subjects were required indicate their level of agreement to each of the questionnaire sentences by putting a mark on a 14 cm-long horizontal line (left-most edge: “totally disagree”, right-most edge: “totally agree”, centre: “neither agree nor disagree”). Twenty-eight items referring to the three phases of the experiment (pre-observation, observation, post-observation) were administered (see Appendix S1). The questionnaire assessed for possible difficulties encountered while performing the tasks, the subjects' perception of wrongness in the observed movements, the feeling of ownership (e.g., it felt as if the moving hand was mine) and agency (e.g., it felt as if I could control the moving hand) about the examiner's hand. The last part of the questionnaire compared subjects' performance before and after the error observation and the subjective perception of any problems potentially caused by the goggles.

#### Statistical analysis

Similar analyses as in Experiment 1 (ANOVAs, t-test) were performed to compare the subjects' performance in PS, VS and VPS following the exposure phase and to additionally verify the presence of groups' differences in the questionnaire.

### Results

**Figure 5:**
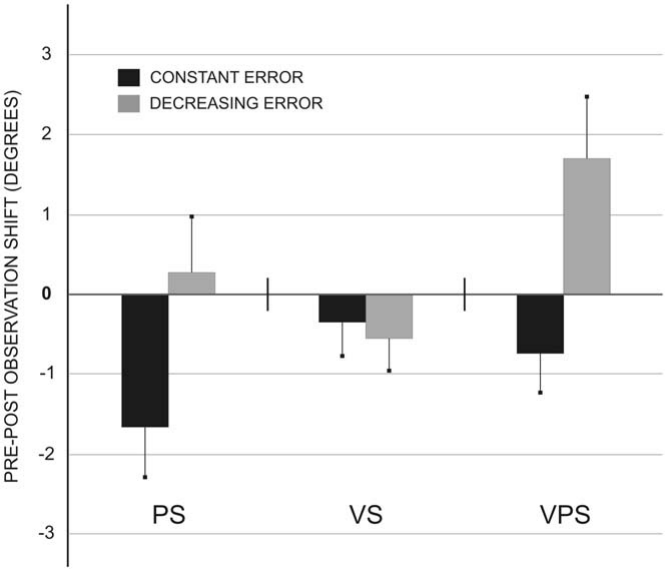
The mean (s.e.m.) proprioceptive (PS), visual (VS) and visual-proprioceptive (VPS) shift in the two groups (Experiment 2). The number zero indicates the lack of differences between pre- and post-observation, negative numbers the presence of a shift to the left, positive numbers to the right.

A preliminary between-subject ANOVA revealed no differences between naive and non-naive group performances in any of the measures. Data were therefore collapsed across groups for the following analyses. Figure 5 illustrates the mean shift for each test as a function of each error observation condition. An ANOVA with Observation (Constant Error, Decreasing Error) as between-subject variable and Shift (PS, VS, VPS) as within-subject variable was performed on mean subjects' performance. The main effect Observation was significant (F (1, 42) = 6.63, p = 0.014), showing that the Constant Error group presented a global leftward shift (PS: −1.66°, VS: −0.35°, VPS: −0.75°) whereas the Decreasing Error group presented, on average, a rightward deviation (PS: 0.26°, VS: −0.56°, VPS: 1.70°). Also the Observation by Shift interaction was significant (F (2, 84) = 3.30, p = 0.042): Fisher post-hoc test confirmed the groups differed with respect to PS (p<0.05) and VPS (p<0.05), but not when the VS was considered.

Complementary analyses were conducted separately for each group by performing t-tests against zero on the three shift measures. In the Constant Error group the PS measure (T = −2.67, p = 0.014) deviated significantly leftward whereas the VS (t = −0.82, p = 0.421) and VPS measure (t = −1.53, p = 0.140) did not show any significant deviation. In the Decreasing Error group, neither the PS (t = 0.38, p = 0.711) nor VS (t = −1.39, p = 0.180) differed from zero, but the VPS measure resulted significantly deviated rightward (t = 2.18, p = 0.041).

#### Questionnaire

**Figure 6:**
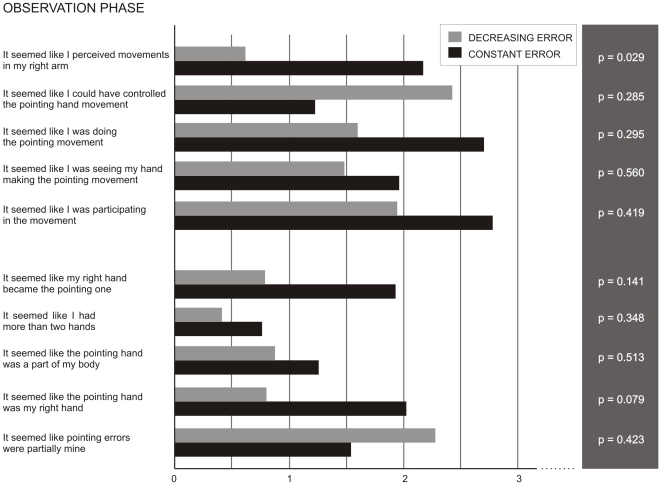
The mean scores (range 0–14) of Constant Error and Reduced Error subjects in some items of questionnaire, related to the presence of agency (above) and ownership (below) of the examiner's hand. On the right, the p-values of unpaired T-tests used to compare the groups' evaluations.

The two groups had similar feelings concerning the first (before observation) and the last (after observation) phases of the experiment. Concerning the observation phase, all subjects identified the presence of incorrect pointing movements (mean level of agreement item 4 = 13.33 for the Constant Error and 13.10 for the Decreasing Error group). Debriefing further confirmed the wrongness was to be attributed to the final landing position not being over the target spot. In addition, subjects in the Constant Error group experienced a stronger illusion to perceive movements in their right arm during the observation phase (mean: 2.17), as compared to subjects in the Decreasing Error group (mean: 0.62; t = 2.26, p = 0.029). As can be seen in Figure 6, several other items referring to the sense of agency and ownership of the experimenter's hand presented the same tendency, albeit not significant, the Constant Error group being more likely to feel participating in the movement and possessing the examiner's hand as compared with the Decreasing Error group.

### Discussion

The results of the second experiment replicate and extend the presence of sensori-motor after-effects following the mere observation of pointing errors. While the possible adaptation-like nature of such after-effects will be addressed in the general discussion below, here we will focus on how the findings of Experiment 2 clarify the points left open by Experiment 1. First, all the participants reported the wrongness of the examiner's pointing movements, appreciating the fact that correct movements have to land on, and not near, the target. Moreover, as shown by comparable levels of agreement in the questionnaire, all the subjects considered the experimenter' pointing movements as incorrect, irrespective of whether they observed the constant or reduced type of error. This finding proved the procedure adopted in Experiment 2 efficient in clarifying to subjects what was meant by “error” in the observed movements, and rules out potential confounds in the interpretation of the difference between pre- and post-observation phases.

Second, at variance with the previous experiment, Experiment 2 explicitly controlled for the possible influence of the participants' previous knowledge about the sensori-motor effects induced by prism adaptation. It might be argued that top-down knowledge could influence subjects' strategy during the observation phase. This may act, in principle, in two ways: reducing the following manifestation of after effects (if subjects oppose to error compensation), or amplifying the after-effects (if subjects favour the error compensation). Therefore, in Experiment 2 subjects who had experienced these effects and had theoretical knowledge about them were contrasted with subjects who were totally naives as to the prism properties and effects. The results showed that neither reduction nor amplification occurred. Naive and non naive subjects had comparable performance, both in the Decreasing and Constant Error condition. Therefore, the leftward after-effect found in PS after observation of pointing errors is not driven by previously acquired expertise about prisms, but it is rather elicited in a bottom-up fashion, by the visual feedback about somebody else's error and by the necessity (or not) to compensate for it.

Another important finding from Experiment 2 was that observation of a constant pointing error induced stronger sensations of movement in the (still) hand of the participants as compared to observation of a progressively decreasing error. While a specific item (“It seemed like I perceived movements in my right arm”) significantly differentiated these conditions, the same tendency was numerically visible in several of the questionnaire's items relating to sense of ownership of the experimenter's moving hand. The illusion that movements of the experimenter hand were felt in the participants' hand was stronger in the Constant than in the Decreasing Error condition, confirming the importance of the subject's cognitive interpretation during the exposure procedure originally proposed by Welch [50]. In that study, participants performed a series of pointing movements towards a target while wearing a pair of goggles; at the end of the movement, they saw the examiner's finger placed leftward relative to their real finger position. When subjects were lead to believe that the goggles induced a lateral visual displacement, they assumed that the finger they saw was their own, and corrected the following pointing movement: in this condition, adaptation (i.e., after-effect) was larger compared to the condition in which participants correctly considered that the finger belonged to the examiner. This is particularly relevant when considering the main result of the present study, namely, that looking at somebody else's erroneous movements engenders compensatory changes in the observers' egocentric reference frames.

#### General Discussion

The present findings provide the first evidence that mere passive observation of erroneous pointing movements can overtly affect sensori-motor coordination in healthy participants. In the first experiment, subjects presented leftward shifts in the proprioceptive and visual estimation of their body midline after the observation of constant, rightward pointing errors. In the second experiment, whereby visual exposure was limited to the second and third part of the pointing movements as in regular prism adaptation, we largely replicated and clarified this pattern: when comparing after-effects emerging from two different type of error signals (constant vs. decreasing), only subjects who observed a constant error presented an after-effect in the opposite (leftward) direction in the proprioceptively based measure (i.e., PS). The results of experiment 2 made also clear that these effects are likely to rely on low-level sensori-motor processing, as they were completely immune to the previous theoretical and/or practical experience with the effects induced by prisms exposure.

In the domain of prism adaptation, and in keeping with Held's principle of reafference, the presence and visibility of the terminal error is thought of as essential to implement error-corrective motor responses and to have consequent after-effects [11]–[13], [51], [52]. The importance of the final pointing error during prism exposure is strengthened by the well-established finding that after-effects are much larger when subjects point to precisely localised targets as compared to when they make movements without a specific target, a condition which provides much poorer feedback about spatial accuracy (see [1] for a review). Another factor influencing prism adaptation is the type of movement exposure: even if it has been proposed that it is possible to marginally adapt also when the arm is moved passively, the greatest adaptation is typically generated when subjects perform active pointing movements, because more discordance between visual and proprioceptive information is produced in the case of self-generated pointing movements [1], [2], [51]–[53]. Finally the importance of proprioceptive information during adaptation processes is not completely clarified: even if some evidence contrast with the possibility to adapt without proprioceptive feedback, it has been demonstrated that deafferented subjects can show visuomotor adaptation, even if considerable cognitive resources were required to strategically control reaching movements [54].

Remarkably, here we demonstrate that passive observation of rightward terminal errors of pointing movements is sufficient to generate a leftward shift of the proprioceptive straight-ahead. In addition, our results emphasise that the type of visual feedback (Constant vs. Decreasing error) may be crucial in producing the pattern of after-effects we observed. The Proprioceptive Shift, one of the most accepted indexes used to measure the presence of prism adaptation after-effects, was clearly sensitive to the presence of a constantly biased feedback provided during the observation phase, with the consequent leftward shift in the proprioceptive straight-ahead pointing (PS). In line with our hypothesis, this finding proves that observation of this type of error elicits some kind of “sensorimotor compensation”. A possible explicative model is suggested. When subjects perform a reaching movement, feedback information about the outcome of the action are integrated in the movement control loop and used to modulate the motor command [55]. On the contrary, during the observation condition subjects are unable to perform any action: this constraint can activate not real, but only desired plans of movement, that are fed by continuous visual feedbacks about failed reaching acts. This discrepancy between intended outcome and real (observed) movement may trigger opposite after-effects in the attempt to correct for the error. Note that, compared to regular exposure to (e.g., rightward) prisms, in our experiments only the hand movements require to be compensated (leftward) for the seen error, as the observer's eyes are not deviated (rightward). The PS is therefore the most appropriate index of adaptation-like mechanisms. In this situation, our model predicts that the PS should be affected (leftward), but the VS should not, because of the lack of any conflicting reafference from the eyes. As a corollary, the model also predicts no summation of effects to result in the VPS measure. Results of Experiment 2 clearly support these predictions: only a “proprioceptive-motor” compensation of the movement was obtained in the direction that would be expected from a rightward displacing prism (i.e., PS leftward). In Experiment 1, a significant visual shift was additionally obtained, but it would hardly be attributable to adaptation-like mechanisms, in that it was opposite to normal compensatory visual after-effects (i.e., leftwards instead of rightwards). As this effect disappeared in Experiment 2, it might be attributed to the full vision of the pointing arm that was available in Experiment 1, but not in Experiment 2, whereby only the final second part of the movement was visually available. We might speculate that when arm vision was fully available, observers tried to visually intervene over the course of the action and pull the actor's hand towards the target, i.e. toward the left. While this possibility awaits for further clarification, the results from both experiments highlight the PS is the most consistent and reliable index for observation-induced after-effects and support our suggestion that similar principles govern the compensation of seen and enacted pointing errors.

The effect we documented share some similarities with the one that usually occurs during the real prismatic adaptation, when both visual and proprioceptive information are available and conflicting: the novelty of this study is the demonstration that by maintaining only one out of two components normally implicated in the prism adaptation (i.e., movement vision), we can evoke a proprioceptive-motor after-effect in the direction opposite to the seen error. Accordingly, a stronger illusory sensation of movement was evoked by the Constant, compared to the Decreasing, error observation condition. The findings of both experiments converge in showing that when a constant rightward error is observed, an opposite leftward shift results afterwards in the proprioceptive measure of subjective straight ahead. This, in turn, implies that visual exposure to a constant error in somebody else's pointing movements has behavioural consequences for the computation of the egocentric references frame of the observer.

Moreover, the results from experiment 1 and 2 indicate that whether a sensori-motor leftward after-effect is observed or not depends more upon the type of error (constant vs. decreasing) than its amplitude (2 vs. 6 cm in Exp. 1 vs. 2, respectively). Indeed, the amplitude of the smallest final error in the decreasing error condition (2 cm) was comparable to the amount of constant error in Experiment 1 (2 cm, constant). Despite this similarity in terms of absolute values, the condition in which subjects observed the error gradually resolving (from 6 to 2 cm) revealed opposite results. Subjects who observed a decreasing error did not exhibit leftward after-effects and, rather displayed a rightward Visual-Proprioceptive Shift (+1.70°), that is, in the same direction as the seen error. This latter finding clearly indicates that different processes may be involved when observing somebody else's erroneous movements, which depend upon the kind of visual feedback provided. We suggests that the Decreasing error condition does not elicit a strong pressure for compensating for the error, because the examiner indeed corrects the pointing error on a trial-by-trial basis. In this condition, the error resolves spontaneously, and the observer does not have to make any sensori-motor “effort” to try to reduce errors. Accordingly, observers were less inclined to attribute the pointing movement to themselves, as compared to the Constant error condition. There was therefore a smaller conflict between the seen and the ideal movement, which might be critical for the emergence of adaptation-like after-effects (i.e., opposite to the erroneous visual feedback given to the participant).

Still, the observation of a Decreasing error did not just produce a null result, as the Visual-Proprioceptive measure of straight ahead was actually shifted, but in the same (rightward) direction of the seen error. Relevant in this respect are recent works by Dupierrix, Alleysson, Ohlmann, and Chokron [56] who reported that a lateralized visuo-motor activity can induce a modification in space perception: after a short session in which subjects were simply required to point to the right of their body midline, Dupierrix and colleagues found a rightward shift in a line bisection task, the opposite (leftward) effect being present after a pointing session towards the left. Interestingly, the same lateralised pointing activity was also shown to modulate the subjective perception of the straight-ahead [57]. In the present study, although the examiner gradually reduced the visible error, the correction only brought the errors down to about 2 cm rightward from the target. We therefore suggest that the results we obtained in the Decreasing error condition reflect a change in the reference frame that is produced by observing sustained lateralised pointing activity towards external targets. In this case, the reference frame is deviated in the same direction (i.e., rightward) of the seen error. Note that the lateralized pointing movements affect only the VPS and not the PS or VS: this result seems to indicate that we modified an “allocentric” space perception, as the series of pointing errors observed are both in the right and left egocentric hemi-space, but always at the right-side of the visual target.

#### Conclusions

To observe another person making a constant rightward error in pointing movements induces a leftward after-effect in the proprioceptive estimation of the observer's body midline, which bears similarities with the typical after-effects induced by prism adaptation. We suggest that this novel finding can be attributed to sensori-motor “adaptation-like” processes that are put into play to correct for the seen motor error during the observation condition. When this conflict is not present, namely when the error is reduced across trials, the prolonged observation of lateralised pointing movements induces, instead, a visuo-motor bias in the same direction of the seen error. It would be interesting to assess whether neglect patients would be similarly affected by observing somebody else's pointing errors and, in turn, if this may have implications for their rehabilitation [38], [39], [58]–[62].

## Supporting Information

Appendix S1 **Questionnaire.**
(DOC)
