# Supplementary material for: Seeing Your Error Alters My Pointing: Observing Systematic Pointing Errors Induces Sensori-Motor After-Effects
Source: PLoS One. 2011 Jun 23;6(6):e21070. doi: 10.1371/journal.pone.0021070 (PMC3121736; doi:10.1371/journal.pone.0021070)
Supplement: Appendix S1 — Questionnaire. (DOC) [file pone.0021070.s001.doc]

*Before wearing the goggles*

1. No problem occurred during the exercise.
2. The exercise was complex .
3. The target was well visible.
4. It was easy to point.

*During wearing the goggles*

1. I perceived something strange in the pointing hand.
2. If I were executing the pointing movements, my movements would have been realized in the same way compared to what I saw.
3. It seemed like I was participating in the movement.
4. I perceived some errors during the pointing movements.
5. It seemed like pointing errors were partially mine.
6. It seemed like I was seeing my hand making the pointing movement.
7. It seemed like the pointing hand was my right hand.
8. It seemed like the pointing hand was a part of my body.
9. It seemed like I had more than two hands.
10. It seemed like my right hand became the pointing one.
11. It seemed like I was doing the pointing movement.
12. It seemed like I could have controlled the pointing hand movement.
13. It seemed like I perceived movements in my right arm.
14. It seemed like the goggles were specials.

*After wearing the goggles*

1. No problem occurred during the exercise.
2. The exercise was complex.
3. The target was well visible.
4. It was easier to point.
5. It was more difficult to point.
6. It seemed like I was doing pointing errors.
7. It seemed like I was pointing more on one side.
8. It seemed like my right hand was attracted toward one side.
9. Some people can have some sight problems caused by the goggles: this happened also to me.
10. Some people can have some pointing problems caused by the goggles: this happened also to me.
